# Application of diffusion microstructure imaging in musculoskeletal radiology *—* translation from head to shoulders

**DOI:** 10.1007/s00330-022-09202-7

**Published:** 2022-10-29

**Authors:** Alexander Rau, Pia M. Jungmann, Thierno D. Diallo, Marco Reisert, Elias Kellner, Michel Eisenblaetter, Fabian Bamberg, Matthias Jung

**Affiliations:** 1 Department of Diagnostic and Interventional Radiology, Medical Center, University of Freiburg, Faculty of Medicine, University of Freiburg, Breisacher Str. 64, 79106 Freiburg, Germany; 2 Department of Neuroradiology, Medical Center, University of Freiburg, Faculty of Medicine, University of Freiburg, 79106 Freiburg, Germany; 3 Medical Physics, Department of Diagnostic and Interventional Radiology, Medical Center, University of Freiburg, Faculty of Medicine, University of Freiburg, 79106 Freiburg, Germany; 4 Department of Stereotactic and Functional Neurosurgery, Medical Center, University of Freiburg, Faculty of Medicine, University of Freiburg, 79106 Freiburg, Germany

**Keywords:** Magnetic resonance imaging, Muscle fibers, skeletal, Rotator cuff, Diffusion tensor imaging, Diffusion magnetic resonance imaging

## Abstract

**Objectives:**

Quantitative MRI techniques, such as diffusion microstructure imaging (DMI), are increasingly applied for advanced tissue characterization. We determined its value in rotator cuff (RC) muscle imaging by studying the association of DMI parameters to isometric strength and fat fraction (FF).

**Methods:**

Healthy individuals prospectively underwent 3T-MRI of the shoulder using DMI and chemical shift encoding-based water-fat imaging. RC muscles were segmented and quantitative MRI metrics (V-ISO, free fluid; V-intra, compartment inside of muscle fibers; V-extra, compartment outside of muscle fibers, and FF) were extracted. Isometric shoulder strength was quantified using specific clinical tests. Sex-related differences were assessed with Student’s *t*. Association of DMI-metrics, FF, and strength was tested. A factorial two-way ANOVA was performed to compare the main effects of sex and external/internal strength-ratio and their interaction effects on quantitative imaging parameters ratios of infraspinatus/subscapularis.

**Results:**

Among 22 participants (mean age: 26.7 ± 3.1 years, 50% female, mean BMI: 22.6 ± 1.9 kg/m^2^), FF of the individual RC muscles did not correlate with strength or DMI parameters (all *p* > 0.05). Subjects with higher V-intra (*r* = 0.57 to 0.87, *p* < 0.01) and lower V-ISO (*r* = −0.6 to −0.88, *p* < 0.01) had higher internal and external rotation strength. Moreover, V-intra was higher and V-ISO was lower in all RC muscles in males compared to female subjects (all *p* < 0.01). There was a sex-independent association of external/internal strength-ratio with the ratio of V-extra of infraspinatus/subscapularis (*p* = 0.02).

**Conclusions:**

Quantitative DMI parameters may provide incremental information about muscular function and microstructure in young athletes and may serve as a potential biomarker.

**Key Points:**

*• Diffusion microstructure imaging was successfully applied to non-invasively assess the microstructure of rotator cuff muscles in healthy volunteers.*

*• Sex-related differences in the microstructural composition of the rotator cuff were observed.*

*• Muscular microstructural metrics correlated with rotator cuff strength and may serve as an imaging biomarker of muscular integrity and function.*

## Introduction

Quantitative MRI techniques are increasingly applied to non-invasively assess the structural integrity of muscles. Chemical shift encoding-based water-fat MRI allows the evaluation of the degree of muscle fatty infiltration (fat fraction, FF) and may play a role in the context of diagnosis and management of degenerative diseases, including planning and outcome assessment of surgical therapies [1]. More recently, several quantitative MRI techniques have been evaluated for trauma and therapy monitoring of muscle injuries in athletes. T2 relaxation time measurements have been applied for the assessment of edema development in delayed-onset muscle soreness (DOMS) [2, 3]. Intravoxel incoherent motion (IVIM) MRI has been used for measurement of microvascular muscle perfusion and visualization of muscle activation in walking and running [4].

Due to the highly organized macro- and microstructure, a directional diffusivity similar to that of neuronal axons is found in muscle [5]. As the sizes of myofibrils (1–2 μm) and neuronal axons (0.01 to 10 μm) are in a similar range [6, 7], diffusion-based MRI (dMRI) techniques such as diffusion tensor imaging (DTI) may provide additional information on structural and microstructural alterations of skeletal muscle before they become visible on morphologic MRI [8–10]. DTI metrics provide insight into the microstructure as they reveal subvoxel information in a mesoscopic approach [11, 12]. Elevated diffusivity was described in inflamed muscle, and reduced diffusivity and less anisotropic diffusion were found in muscles with fatty infiltration [13, 14]. Moreover, DTI has been applied to assess muscle tears in athletes [15, 16].

Recently, advanced multicompartment techniques like diffusion microstructure imaging (DMI) have provided substantial additional value in neuroimaging and allowed for even more specific insights into the microstructural integrity of tissue compared to DTI [17–20]. Rather than only providing information on the directionality and orientation of the diffusivity within a voxel as in DTI, DMI approximates the distribution of three microstructural compartments per voxel.

For the quantitative assessment of rotator cuff (RC) muscles, advanced quantitative MR imaging techniques are of major interest [1, 12, 21–23]. Quantitative muscle MR measurements may be important for treatment decisions with respect to the operative treatment technique [1, 21]. In young, active patients, decentering of the shoulder often occurs due to weakness of the external rotators. These patients require strengthening of the RC to address external rotation strength imbalances for injury prevention [24–26]. MRI may help to detect and monitor such insufficiencies and contribute to evaluate physical therapy success and decision-making on return-to-play in athletes.

Therefore, the purpose of this study was (i) to demonstrate the feasibility of DMI of RC muscles and provide an initial estimate of normal values, (ii) to assess the association with MR imaging-based FF measurements, and (iii) to correlate DMI with isometric strength measurements in healthy volunteers.

## Material and methods

### Study participants

This prospective study was approved by the local Institutional Review Board (EK:1446/21). All procedures performed in studies involving human participants were in accordance with the ethical standards of the institutional and national research committee and with the 1964 Helsinki declaration and its later amendments. Informed consent was obtained from all individual participants included in the study. Exclusion criteria were age < 18 years, previous shoulder surgery, and neurologic, or muscular disorders.

### Magnetic resonance imaging

MR imaging of the right shoulder was performed at a 3.0 T MR scanner (Magnetom Vida, Siemens Healtineers) using a dedicated 16-channel shoulder coil (Shoulder Shape 16, Siemens Healtineers). The arm was positioned in neutral zero position. For chemical shift encoding-based water-fat separation of the RC, a transverse-prescribed two-point T1w Dixon sequence was acquired with the following parameters: repetition time (TR) = 4.2 ms, echo times (TE) = 1.23 and 2.46 ms, flip angle = 5°, GRAPPA factor = 2, voxel size = 0.7 × 0.7 × 3.0 mm^3^, phase encoding direction = A/P, consecutive slices = 48, scan time = 54 s. The dMRI sequence was adapted from the previously published brain-specific sequence [19] to address the specific requirements for dMRI in the musculature [27–29] resulting in the following parameters: transverse orientation, 23 consecutive slices, voxel size = 2.0 × 2.0 × 4 mm^3^, TR = 4500 ms, TE = 90 ms, bandwidth = 2222 Hz/Px, GRAPPA factor = 2, 12 diffusion-encoding gradient directions, b-factors = 600 and 1000 s/mm^2^; scan time = 5:35 min.

### Semiquantitative MR analysis

MR images were transferred on Picture Archiving Communication System workstations (Deep Unity, Dedalus HealthCare) and evaluated semiquantitatively by 2 radiologists in consensus with 3 and 7 years of experience in musculoskeletal imaging, respectively. The in-phase Dixon images were used for semiquantitative evaluation of RC muscle atrophy and fatty infiltration. Goutallier score was employed for assessment of fatty infiltration [30]. The Cofield score was used for semiquantitative evaluation of muscle atrophy [31]. Additionally, the presence of partial or complete RC tear was excluded on standard clinical proton density-weighted fat-saturated sequences which were assessed in transverse, coronal, and sagittal orientation.

### Quantitative MRI parameter calculation

To investigate the fat-water composition of the RC muscles, the fat fraction (FF) was derived from the Dixon sequence as the ratio of the fat signal over the sum of fat and water signals. The DMI parameters of the RC muscles were calculated within our in-house post-processing platform NORA (www.nora-imaging.org). Pre-processing of diffusion-weighted images included a denoising step [32] followed by correction of the Gibbs-ringing artifacts [33] and upsampling to an isotropic resolution of 2.0 mm^3^. Microstructural diffusion metrics were estimated using a Bayesian approach [34] and three volume fractions were determined adapted from the brain’s white matter standard model: (1) the free water fraction (V-ISO) in that molecules randomly move at the distance of their diffusion length (in the range of a tenth of micrometers); (2) the volume fraction within muscular fibers with almost one-dimensional molecule diffusion due to tight membrane borders of the sarcolemma (V-intra); (3) the volume fraction outside of muscular fibers (V-extra) is characterized by an intermediate constraint to molecule diffusion representing intramuscular connective tissues such as endo- and perimysium.

### Volume of interest (VOI)–based analysis

**Fig. 1:**
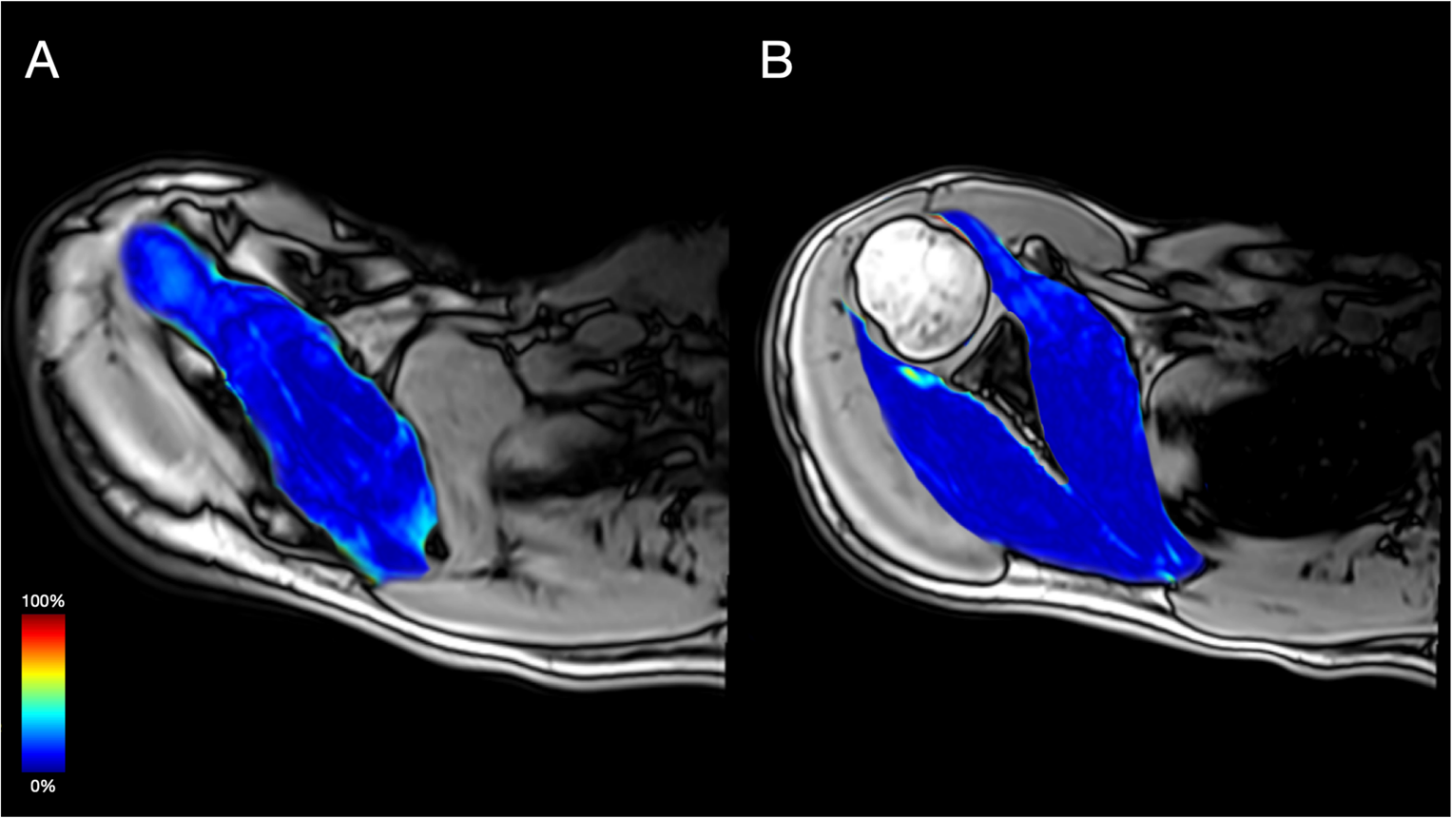
Exemplary representation of the manually performed segmentation of the rotator cuff muscles. Calculated fat fraction color maps overlaid onto the opposed-phase of T1w Dixon. **A** SSP, (**B**) SSC and ISP. Cold color indicates low FF, and hot color indicates high FF

Supraspinatus (SSP), subscapularis (SSC), and infraspinatus (ISP) were manually segmented on the opposed-phase Dixon sequence along the muscular borders by one trained radiologist (3 years of experience in musculoskeletal radiology, Fig. 1). Tendinous structures and the investing fascia were carefully excluded from segmentation. VOIs were subsequently overlaid on FF maps and on the *b* = 0 images to approve correct alignment. DMI-derived parameters and FF were extracted from the VOIs.

### Isometric strength measurements

Shoulder abduction, internal rotation, and external rotation isometric strengths were measured (in kilogram) bilaterally by using an IsoForceControl Evo2 dynamometer (MDS Medical Device Solutions) with 33 measurements per second for a 5-s period. The dynamometer cuff was positioned just proximal to the wrist. For SSP function, strength testing of abduction was assessed with the participant in a seated position and the arm at 90° of elevation in the scapular plane, and the forearm in pronation (Jobe test position). In addition, a strength measurement with the patient in a standing position and the arm in 0° abduction was obtained (Starter test position). For ISP and SSC analyses, measurements of internal and external rotation strength were performed at 0° of abduction, maintaining the elbow in 90° flexion and the wrist at neutral position. The mean and maximum value within the 5-s period of the second measurement of each shoulder was used for data analysis. The ratios of the mean and maximum strength of external/internal rotation (ER/IR) were calculated [25].

### Statistical analysis

Shapiro–Wilk test was used to assess the normal distribution of data. Sex-related differences in DMI-parameters, FF, and strength between male and female participants were assessed with Student’s *t*. Pearson’s and partial Spearman’s (controlling for sex) correlation were employed to relate DMI-metrics, FF, and strength. To investigate microstructural correlates of muscular dysbalance in the RC, we split our cohort with an ER/IR strength ratio cutoff of 0.95 [25, 35–37]. Similar to the ER/IR strength ratio, we calculated a corresponding ratio of the quantitative MRI parameters of ISP/SCC. A factorial two-way ANOVA was performed to compare the main effects of sex and ER/IR strength ratio and their interaction effects on quantitative parameters ISP/SSC ratios. Continuous parametric variables are reported as median and range or mean and SD, and values with an *α*-level of 0.05 were considered statistically significant. Since the analyses were mainly explorative, *p*-values were not adjusted for multiple testing.

All statistical analyses were performed using R statistics (R-3.5.3 – R Core Team, https://www.R-project.org).

## Results

### Study participants

A total of 22 healthy volunteers (mean age 26.7 ± 3.1 years; 11 male, 11 female) were included in this study. There was a statistically significant difference for male and female participants in BMI (23.6 ± 1.8 kg/m^2^ vs. 21.6 ± 1.5 kg/m^2^; *p* = 0.01) but not in age (26.3 ± 1.8 years vs. 27.1 ± 3.5 years; *p* = 0.55).

### Strength measurements

Strength measurements revealed statistically significant higher values for mean and maximum strength for the Jobe test, starter test, internal rotation, and external rotation in males (all *p* = < 0.001; Table 1). Ipsilateral and contralateral strength and ER/IR strength ratios were not different (all *p* > 0.05). Ipsilateral and contralateral strength correlated significantly (all *p* < 0.001).

**Table 1:** Participant characteristics, quantitative imaging features, and clinical readouts

		Male		Female		Sex-difference
		Mean	SD	Mean	SD	*p*-value
Characteristics	Age (years)	26.30	2.76	27.10	3.45	0.55
	Weight (kg)	78.70	5.08	63.20	4.92	< .001
	Height (m)	1.83	0.06	1.71	0.04	< .001
	BMI (kg/m^2^)	23.60	1.81	21.60	1.48	0.01
Subscapularis	V-intra	0.42	0.04	0.29	0.05	< .001
	V-extra	0.27	0.01	0.28	0.01	0.248
	V-ISO	0.32	0.04	0.44	0.05	< .001
	FF	2.17	0.51	9.29	15.50	0.14
Infraspinatus	V-intra	0.28	0.05	0.21	0.05	0.006
	V-extra	0.30	0.02	0.29	0.04	0.704
	V-ISO	0.42	0.04	0.49	0.08	0.012
	FF	5.00	8.97	6.62	12.50	0.73
Supraspinatus	V-intra	0.45	0.07	0.38	0.06	0.022
	V-extra	0.27	0.02	0.28	0.02	0.156
	V-ISO	0.53	0.06	0.34	0.05	0.015
	FF	14.70	14.00	21.60	23.80	0.46
Internal Rotation	Mean	11.40	1.28	6.53	0.93	< .001
	Maximum	12.80	1.61	7.90	1.05	< .001
External Rotation	Mean	10.40	1.90	5.58	1.19	< .001
	Maximum	11.90	1.67	6.37	1.18	< .001
Jobe test	Mean	11.30	2.43	7.23	1.21	< .001
	Maximum	12.80	3.31	8.18	1.39	< .001
Starter test	Mean	11.10	2.84	6.82	1.45	< .001
	Maximum	12.30	2.71	7.80	1.56	< .001
ER/IR strength ratio	Right mean	0.94	0.16	0.99	0.13	0.36
	Right maximum	0.96	0.13	0.95	0.09	0.83
	Left mean	0.93	0.16	0.91	0.19	0.77
	Left maximum	0.94	0.14	0.87	0.17	0.35
ISP/SSC ratio	V-intra	0.67	0.09	0.74	0.17	0.242
	V-extra	1.13	0.07	1.08	0.14	0.304
	V-Iso	1.33	0.13	1.15	0.22	0.036
	FF	2.78	5.76	1.06	0.36	0.34

*FF*, fat fraction; *ER*, external rotation; *IR*, internal rotation; *ISP*, infraspinatus; *SSC*, subscapularis

### Semiquantitative RC assessment

None of the participants showed muscle atrophy (all Cofield grade 0). Regarding fatty infiltration of the RC muscles, *n* = 8 (5 female) showed Goutallier grade 1. All other participants (*n* = 14) had a Goutallier grade of 0. All volunteers had no RC tear.

### Quantitative MR imaging

**Fig. 2:**
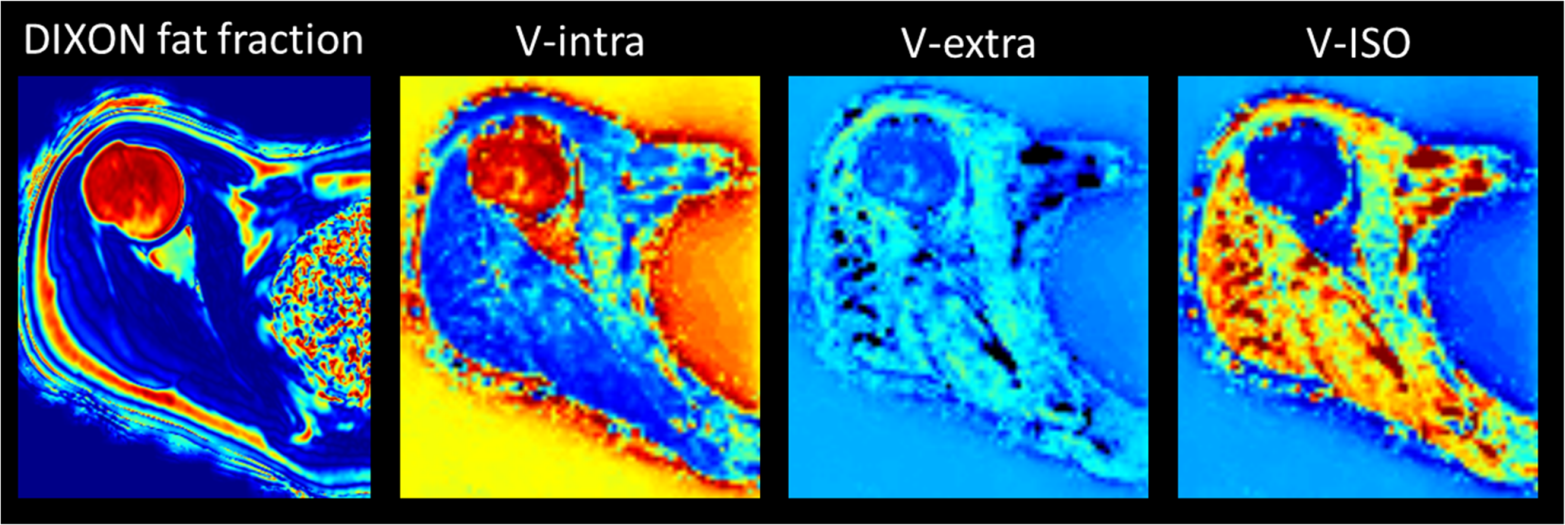
Exemplary representation of the acquired quantitative MRI sequences in a 25-year-old female

Quantitative image parameters were successfully calculated in all subjects (an exemplary case is depicted in Fig. 2). Mean FF for the SSP was 18.5% ± 19.8%, for the ISP 5.8% ± 10.7%, and for the SSC 5.7% ± 11.3%. Although FF was lower in males as compared to females for all analyzed RC muscles, the difference was not statistically significant (mean of all muscles was 6.3% ± 10.3% in males vs. 12.5% ± 18.6% in females, *p* > 0.05; see Table 1).

**Fig. 3:**
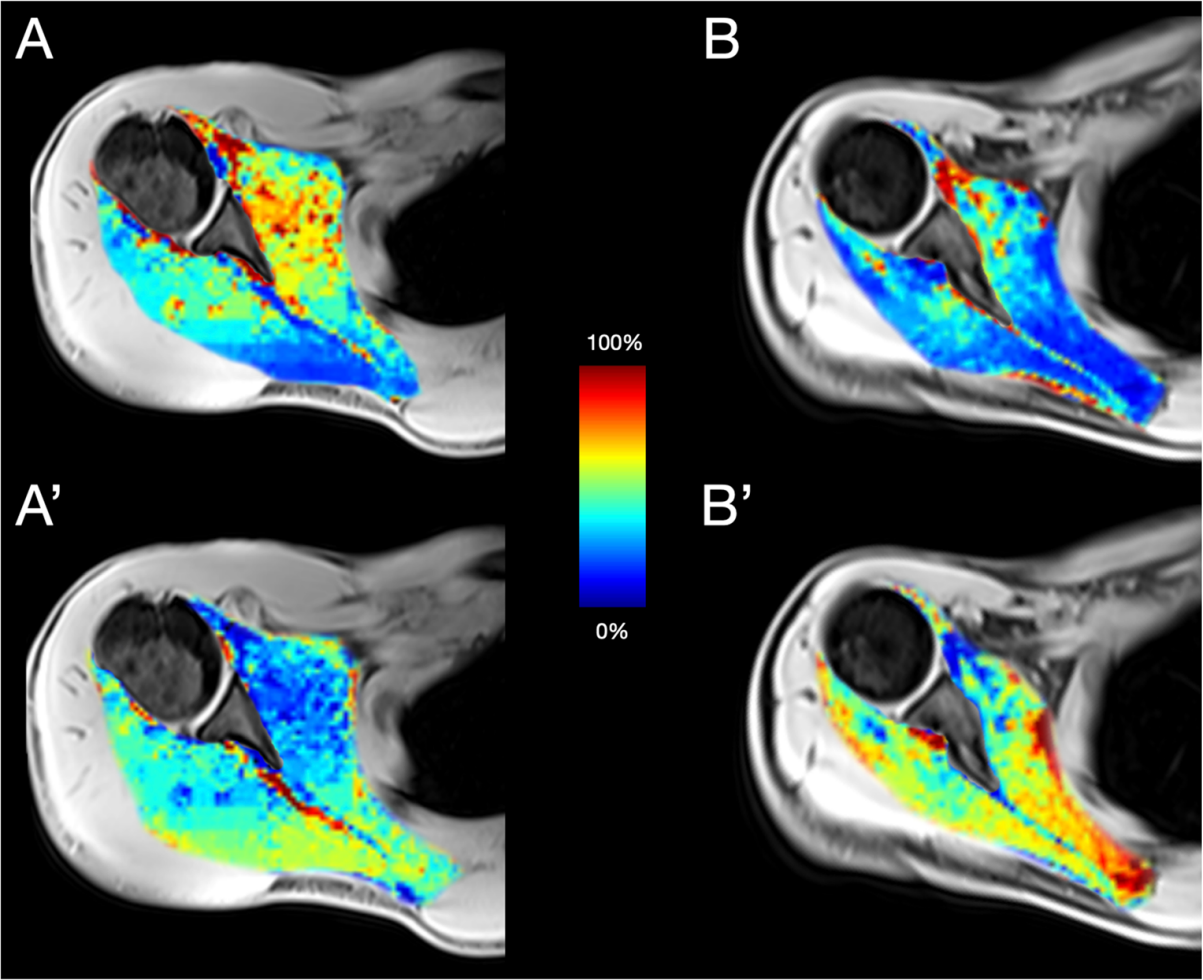
Quantitative DMI parameters of the SSC and ISP in an exemplary male (left column) and female participant (right column). V-intra (**A**, **B**) was higher and V-ISO (**A’**, **B’**) was lower in all RC muscles in male (left column) compared to female subjects (right columns). Calculated V-intra (**A**, **B**) and V-ISO (**A’**, **B’**) color maps overlaid onto the water image of T1w Dixon. Cold color indicates low, and hot color indicates high values

Detailed descriptives of the microstructural DMI parameters V-intra, V-extra, and V-ISO are displayed in Table 1. V-intra was significantly higher in all RC muscles of male compared to female subjects (in contrast, V-ISO was significantly lower in the respective RC muscles of male participants compared to females). No statistically significant sex difference was found for V-extra (all *p* > 0.05). Please see Fig. 3 for some exemplary cases.

### Association of FF and DMI parameters with strength

**Fig. 4:**
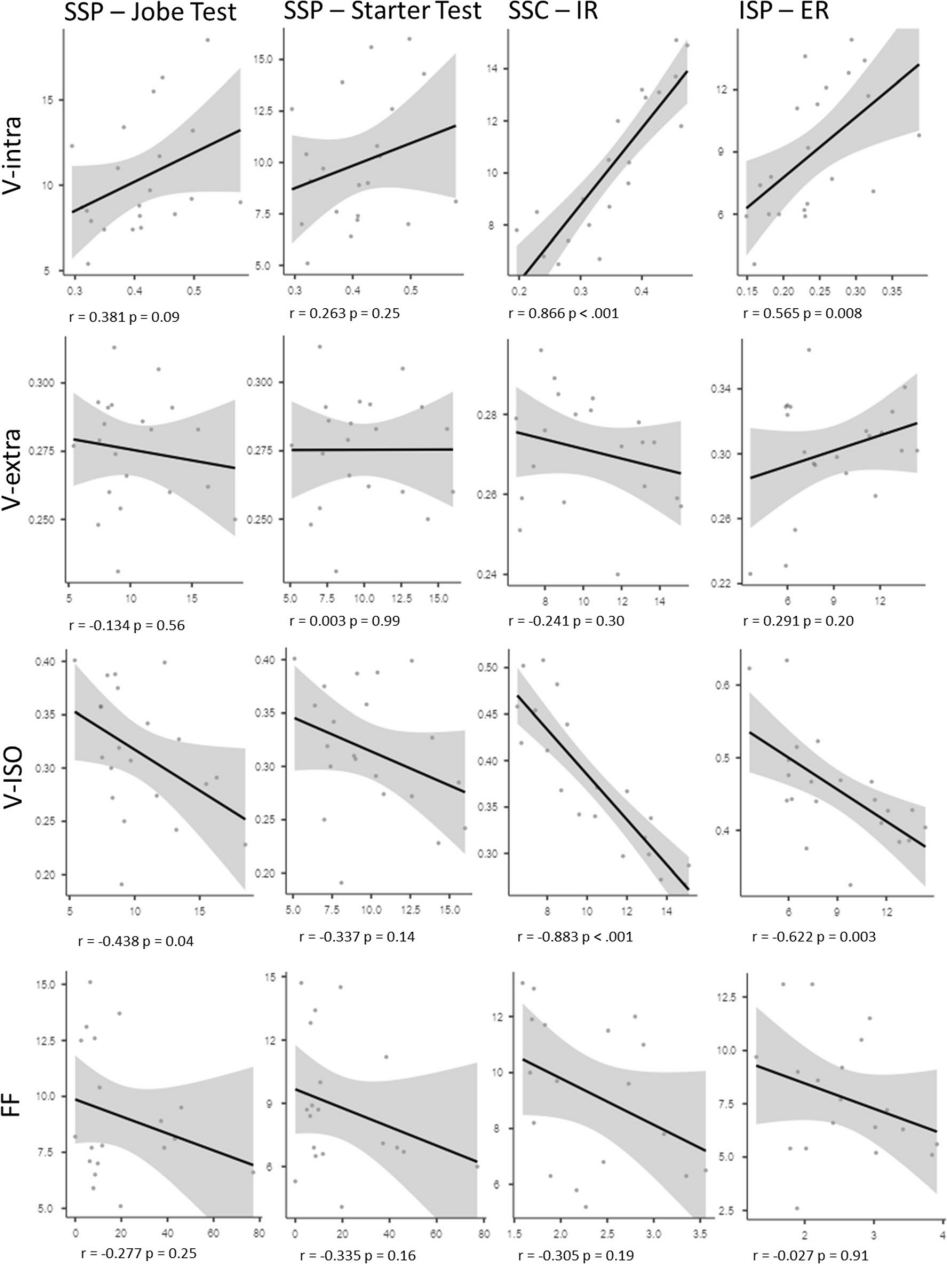
Illustration of the correlations between the DMI parameters and the strength test specific to each muscle. *SSP*, supraspinatus; *SSC*, subscapularis; *ISP*, infraspinatus; *FF*, fat fraction; *IR*, internal rotation; *ER*, external rotation

Mean FF of the individual RC muscles did not correlate with strength or DMI parameters (all *p* > 0.05). A summary of the associations of DMI and FF parameters with strength is shown in Fig. 4. For SSC and ISP, higher V-intra was significantly correlated with higher maximum strength (*p* < .001 and *p* = 0.008). Further, there was a statistical significance for the correlation of lower V-ISO with a higher maximum strength of the SSC (*p* < 0.001), ISP (*p* = 0.003), and SSP (*p* = 0.04).

Controlling for sex, we observed significant associations of SSC V-intra and maximum strength (rho 0.52, *p* = 0.01) and SSC V-ISO and maximum strength (rho 0.65, *p* = 0.003) as well as SSP V-extra and mean strength (rho 0.46, *p* = 0.04).

### Relationship of sex, ER/IR strength ratio, and quantitative MRI parameters

For the V-extra ISP/SSC ratio, the main effect for ER/IR strength ratio yielded an F ratio F(1, 16) = 6.59, *p* = 0.02, indicating a significant difference between the subgroup with an ER/IR ratio > 0.95 vs. the subgroup with a ratio < 0.95. The main effect of sex yielded an F ratio F(1, 16) = 0.11, *p* = 0.74, indicating that the effect of sex was not significant. The interaction of sex and the ER/IR strength ratio was not significant (*p* = 0.66). No significant ER/IR strength ratio differences were observed for V-intra, V-ISO, or FF.

## Discussion

In this study, we have used specifically adapted, advanced quantitative MRI techniques to assess the correlation between fat-water and microstructural composition of RC muscles and their associations with muscle strength in young healthy individuals. We found significant correlations between DMI parameters and the mean and maximum strength of the respective RC muscles with the strongest effects in V-intra and V-ISO. Further, there were significant sex differences in the DMI parameters V-intra and V-ISO. In contrast, the mean FF of the individual RC muscles did not correlate with individual strength. Therefore, our results indicate that multicompartmental DMI measurements could provide more detailed information about RC muscle composition and function than FF in young healthy individuals.

Preoperative characterization of RC muscle quality is crucial for surgical treatment planning and postoperative outcome. In addition, muscle quality is important for proper function in healthy individuals and athletes. MR imaging may help to detect muscle weakness leading to decentering of the shoulder and may potentially be able to monitor the effects of physical therapy. Increasingly applied quantitative MRI techniques may overcome the current problem of high interobserver variability in classic qualitative MRI [38, 39]. Previous studies showed correlations of RC muscle FF with the semi-quantitative Goutallier score [30, 40]. Further, Karampinos et al showed significant inverse correlations of RC FF with isometric strength measurements 10 years after RC repair in patients with a mean age of 72 years [40]. A similar finding was reported by Davis et al in a mixed study population of patients with and without full-thickness RC tears and a combined mean age of 62.2 years [41]. In contrast, no significant correlation between RC muscle FF and strength was found in the present study. This may be due to the notably younger study population (mean age 26.7 years) without relevant fatty infiltration. Most interestingly, despite the lack of correlations with FF, we observed significant correlations of strength with DMI parameters.

Previously, advanced dMRI techniques have been assessed for potential applications in shoulder imaging. In a case series of two patients after RC tendon repair, Fieremans et al found myofiber size reduction in SSP and ISP using time-dependent DTI measurements and a random permeable barrier model [27]. Moreover, age-associated changes and variances of DTI parameters among visually intact RC have been shown in a cohort of 12 females (mean age 44.3 years) and 28 males (mean age 39.4 years) [12]. The same study reported a low but significant correlation of DTI parameters with FF. In contrast to this, we found no significant correlation between DMI parameters and RC FF. In combination with the fact that we nevertheless found correlations between DMI parameters and RC strength, this suggests that DMI parameters may provide additional, FF-independent information on muscle quality. However, in our cohort of young individuals with a narrow BMI range, most correlations of DMI parameters and RC strength attenuated when controlling for sex. Therefore, larger studies are required to assess influence factors such as sex, age, BMI, and metabolic factors as well as morphological alterations of the RC on DMI parameters. Of note, DMI parameters showed a significant sex difference. Most likely the lacking sex difference of the FF may become evident in a larger cohort, while the difference was already evident for DMI in the analyzed cohort with *n* = 11 subjects of each sex. This finding underlines that DMI may be more sensitive than FF to interpersonal differences in muscle composition.

In neuroimaging, multicompartment dMRI-based approaches were found to offer more specific metrics and to be more sensitive to pathological tissue alterations than single-compartment DTI [18, 42]. Similar assumptions are reasonable for musculoskeletal radiology. Karampinos and colleagues assumed two main compartments contributing to the dMRI signal, i.e., the intracellular (within the muscle fiber) and the extracellular space (collagenous intramuscular connective tissues consisting of endomysium and perimysium) [43]. Tan et al. assessed the fiber diameter in patients with muscle denervation at the shoulder based on the diffusion signal and observed a more robust depiction of the microstructural changes in comparison to DTI [28]. Our DMI approach further considers an additional free fluid compartment (V-ISO). In a previous study, our DMI approach was reported to be highly sensitive to discrete shifts of microstructural compartments otherwise not detectable on conventional MRI [19]. DMI was also more sensitive in detecting pathological changes in brain tissue in the context of neurodegeneration than DTI [18].

Comparable to neuronal axons, the myofiber is a complex, but highly organized structure that is composed of myofibrils. Myofiber hypertrophy is a well-known adaptation of skeletal muscle to repetitive exercise to increase muscle strength and performance [44–46]. In line with this, we here report that higher V-intra and lower V-ISO — which corresponds to a larger proportion of myofibrils per voxel — were associated with higher mean and maximum muscle strength. Therefore, DMI as a novel imaging tool may serve as an imaging biomarker on microstructural integrity or changes in the myofiber fraction before being detectable on conventional MRI. Interestingly, the V-extra ISP/SSC ratio differed significantly between the groups with and without RC external rotation imbalance. DMI may therefore be a valuable tool for the assessment of RC imbalance and non-invasive monitoring of therapy outcomes in athletes with external rotation weakness.

The value of DMI in monitoring physical therapy, as well as evaluating pathological conditions such as trauma, DOMS, or muscle tears, has yet to be evaluated in further prospective studies with larger sample sizes of different age groups. We believe that DMI could serve as an additional non-invasive method to detect early degenerative changes of the RC muscles in elderly patients.

Our study has several limitations. First, our results are limited by the relatively small sample size and the shoulder as the only region investigated, therefore requiring confirmation in larger longitudinal cohort studies. In addition, further adaptation of the DMI model to muscle tissue is needed as the employed Bayesian approach was primarily developed for brain tissue. For this reason, a publicly accessible toolbox is not yet available.

Our MR-based results of FF and DMI measurements were not validated by histopathological correlation which is still considered the gold standard for quantification of fat content and muscle quality assessment. However, previous studies have demonstrated the validity and reproducibility of a standardized MRI-based skeletal muscle FF quantification with good concordance to histology [47].

In this proof-of-concept study, we successfully applied DMI to musculoskeletal imaging. In conclusion, DMI allows for a non-invasive quantitative approximation of muscle microstructure in vivo and may provide further insight into muscular integrity, function, and imbalances in healthy individuals and young athletes.

## Abbreviations

BMI: Body mass index
DMI: Diffusion microstructure imaging
dMRI: Diffusion MRI
DTI: Diffusion tensor imaging
ER: External rotation
FF: Fat fraction
IR: Internal rotation
ISP: Infraspinatus
RC: Rotator cuff
SSC: Subscapularis
SSP: Supraspinatus
V-extra: Compartment outside of muscle fibers
V-intra: Compartment inside of muscle fibers
V-ISO: Free fluid compartment

## Declarations

### Guarantor

The scientific guarantor of this publication is Fabian Bamberg.

### Statistics and biometry

One of the authors has significant statistical expertise.

### Methodology

• prospective

• cross sectional study

• performed at one institution
